# Revolutionizing Fingerprint Detection: Ce^3+^ Doped SiO_2_-Zr_2_O_3_:Sr^2+^ Nanocomposites with Enhanced Luminescence and Selectivity

**DOI:** 10.1007/s10895-024-04028-6

**Published:** 2024-12-06

**Authors:** Momna Qayyum, Sammia Shahid, Sana Mansoor, Muhammad Faizan, Mohsin Javed, Ammar Zidan, Ali Bahadur, Shahid Iqbal, Sajid Mahmood, Abd-ElAziem Farouk, Salman Aloufi

**Affiliations:** 1 Department of Chemistry, School of Science, University of Management and Technology, Lahore, 54770 Pakistan; 2 Department of Chemical and Materials Engineering, Chang Gung University, Taoyuan, 33303 Taiwan; 3 Biomedical Engineering Department, College of Engineering and Technologies, Al-Mustaqbal University, Babylon, 51001 Iraq; 4 Nanomaterials Research Center, Department of Chemistry, College of Science, Mathematics, and Technology, Wenzhou-Kean University, Wenzhou, Zhejiang Province 325060 China; 5 Dorothy and George Hennings College of Science, Mathematics and Technology, Kean University, 1000 Morris Ave, Union, NJ 07083 USA; 6 Nottingham Ningbo China Beacons of Excellence Research and Innovation Institute, University of Nottingham Ningbo China, Ningbo, 315100 China; 7 Functional Materials Group, Gulf University for Science and Technology, Mishref, 32093 Kuwait; 8 Department of Biotechnology, College of Science, Taif University, P.O. Box 11099, Taif, 21944 Saudi Arabia

**Keywords:** Fingerprints, Nano-forensics, Photoluminescence, Sol-gel, Rare Earths

## Abstract

Even though fingerprints remain one of the most reliable methods of identification, they are often lost during the recovery process. Accurate fingerprint recognition depends on the contrast between the ridges and substrate. On tough surfaces, such as glossy, colorful, and patterned materials, the contrast is harder to establish. Photoluminescent materials play a crucial role in forensic investigations as they enable the development of procedures that enhance image quality and increase the accuracy of findings from security institutions. Due to the strong emission in the red area at 620 nm, the use of trivalent Rare Earth ions (RE^3+^) doped materials in this work is notable. Because of the unique properties and abundance of cerium, luminous materials based on SiO_2_-Zr_2_O_3_: Ce^3+^, Sr^2+^ prepared via sol-gel technique present a more practical alternative for use in criminal investigations compared to current photonic materials. The sample was further co-doped with synthetic (Safranin-O and crystal violet) as well as organic (curcumin and lycopene) photoluminescent dyes. The nanocomposites were examined using X-ray diffraction analysis (XRD), energy-dispersive X-ray analysis (EDX), dynamic light scattering (DLS), scanning electron microscopy (SEM), and photoluminescence spectroscopy (PL). In conclusion, this work highlights the qualities critical to obtaining higher-resolution latent fingerprint images for potential forensic applications.

## Introduction

Nano forensics uses nanotechnology to increase the sensitivity and accuracy of recognizing anonymous evidence in forensic science investigations by replacing bulky instruments. It comprises creating state-of-the-art instruments for tasks such as explosive detection, gunshot residue analysis, fingerprint visualization, illicit drug detection, and bodily fluid detection [1, 2]. It provides advanced tools for real-time crime investigations, including nano-imaging, nano-manipulators, and nano-sensors [3–5]. Every person leaves their unique fingerprint pattern on an object the moment they touch it [3], and this pattern does not change over time [4, 5]. These patterns give vital information for victim and suspect identification. These fingerprint imprints often referred to as LFPs, are typically invisible to the naked eye and require special visualization techniques, including powder dusting, cyanoacrylate fumes, and spectroscopic analysis by using FTIR, GC, and MS techniques [6]. However, these techniques have drawbacks, including poor discrimination, low optical contrast, non-biocompatible nature, background intervention, and toxicity [6]. Dye-doped powder enhances the visibility and precision of fingerprints but can also leave stains, only work on porous surfaces, and can contaminate the environment if not handled properly [7–9]. Research suggests using synthetic fluorescent fingerprint dusting powders, with nano-technological advancements enhancing sensitivity and selectivity [10]. Fluorescent nanoparticles provide many superior advantages, including minimal toxicity, high selectivity, great contrast, and significant sensitivity when employed for LFP development [11–13].

Photoluminescent materials are utilized in everyday life to improve the quality of life, as they are adaptable to various scientific domains and provide diverse data for research [14]. Their significance extends to forensic analysis, optical fibers, biosensors and biomarkers [15], image-generating devices, optical amplifiers, lasers, photonics [16], and solar cells [17], among other things. Trivalent rare earth (RE) doped luminescent nanocrystals, which emit light when excited, improve the visibility and detection of forensic evidence, including fingerprints. These materials are in high demand in nanophotonics due to their distinctive luminescence characteristics and potential treatments in display devices, biomedical fields, and forensics [18]. The lifetime of luminescence material used to mark fingerprints is also important, as molecules like amino acids, DNA, proteins, cells, and lipids release light due to the π → π* bonding type within their molecular framework [19]. These compounds have excited state lifetimes with values in the order of ns because transitions are allowed due to the parity (ΔL ≠ 0 – Laporte’s rules) [20]. Materials with longer excited state lifetimes can mark fingerprints, enabling the interference-free signal collection linked to the longest-lasting substance [21]. Because of their electrical transition characteristics and millisecond (ms) lifetime, rare earth element-containing bright materials can resolve fingerprinting problems.

Among the rare earth elements, Ce^3+^’s excited 5d energy levels are sensitive to the coordination environment, meaning that both the chromaticity of the emission and the crystal field’s coordination partners rely on it [22]. This provides for a tunable emission color. Because cerium is more abundant than copper on Earth and can be separated from other lanthanide elements more easily, it is relatively inexpensive [23]. In this work, a luminous material based on SiO_2_-Zr_2_O_3_ co-doped with Ce^3+^ and Sr^2+^ was synthesized, following previous literature studies [24, 25], maintaining the unique characteristics of photoluminescence while offering good chemical and thermal stabilities. As oxides are effective hosts for RE^3+^ ions [26]. The luminous material was then further co-doped with the synthetic dyes Safranin-O [27] and crystal violet [28] as well as with the organic dyes curcumin [29, 30] and lycopene to study the effects of added dopants on the chromaticity of the emission spectra. Dyes alone are not suitable for fingerprint development as they require a carrier to maintain their photoluminescent characteristic and adhere to the fingerprint ridges for fine detailing [30]. RE^3+^ luminescence enhances time-resolved imaging by eliminating background fluorescence and scattered light, making it effective for the forensic detection of LFPs [31]. The material’s characteristics shield it against changes in crystalline structure and loss of optical qualities when exposed to high temperatures at crime scenes. Zr_2_O_3_ shows good thermal stability [32].

The Ce^3+^: Zr_2_O_3_ luminous core offers minor symmetry distortion [33] modifying the crystal structure and aiding energy transfer. This prevents energy losses during non-radiative energy processes when energy is transferred from the host matrix to the Ce^3+^ ion, ensuring sufficient luminescence. SiO_2_ enhances biocompatibility, making the system more suitable for a variety of applications [34] and surface imperfections in Ce^3+^: Zr_2_O_3_ [35], making it appropriate for use in forensic applications. Because the system is transparent in the visible spectrum, examination of luminescence data is not hampered. Using the same preparation method, we discovered in earlier studies that this pathway facilitates the synthesis of metallic oxides, which are then inserted into SiO_2_. Examples of these oxides are Ta_2_O_5_ [36] and Nb_2_O_5_ [16], both of which have the RE^3+^ ion situated in their structure. This structure is intriguing because SiO_2_ is exposed, which allows it to interact with the proteins in the fingerprint to enhance and facilitate its impregnation and eventually provide better and more effective information.

The Sr^2+^ ion’s insertion into the matrix may increase asymmetry at the Ce^3+^ site, generate systemic defects, and maintain chemical stability [33, 37]. Using a solution combustion approach, Jisha et al. (2017) synthesized Tb^3+^-doped GdAlO_3_, which resulted in high-resolution fingerprints, and bright photoluminescence in the green area [38]. For latent fingerprint applications, Wang et al. (2015) synthesized YVO_4_: Eu nanocrystals and LaPO_4_:Ce, Tb nanobelts with excellent contrast, sensitivity, and efficiency [39]. For application on latent fingerprints, Venkatachalaiah et al. (2017) obtained a core-shell based on SiO_2_@Y_2_O_3_:Eu^3+^ with high photoluminescence emission [40]. Arantes et al. (2019) reported the synthesis of TiO_2_ powders doped with Eu^3+^, with the most intense emissions in the red region [41]. Cerium-doped nanophosphors with intense red emissions have also been reported recently [42, 43]. Besides, the sol-gel technique is a chemical method that allows for the preparation, handling, and synthesis of materials at room temperature, while also adjusting reagent concentrations [44].

Considering the practicality of the sol-gel technique [37], this study suggests a quick and practical synthesis of a SiO_2_-Zr2O_3_:Ce^3+^, Sr^2+^ photoluminescence material that is further co-doped with Safranin-O, crystal violet (CV), curcumin, and lycopene, for potential use as a fingerprint marker. The study evaluates the influence of Ce^3+^ and Sr^2+^ ions on the SiO_2_ and Zr_2_O_3_ matrix, and the influence of the added dyes as dopants on the intensity and chromaticity of the emissions. It also analyzes the structural and photoluminescent properties of these materials.

## Experimental

### Chemicals and Reagents

**Fig. 1:**
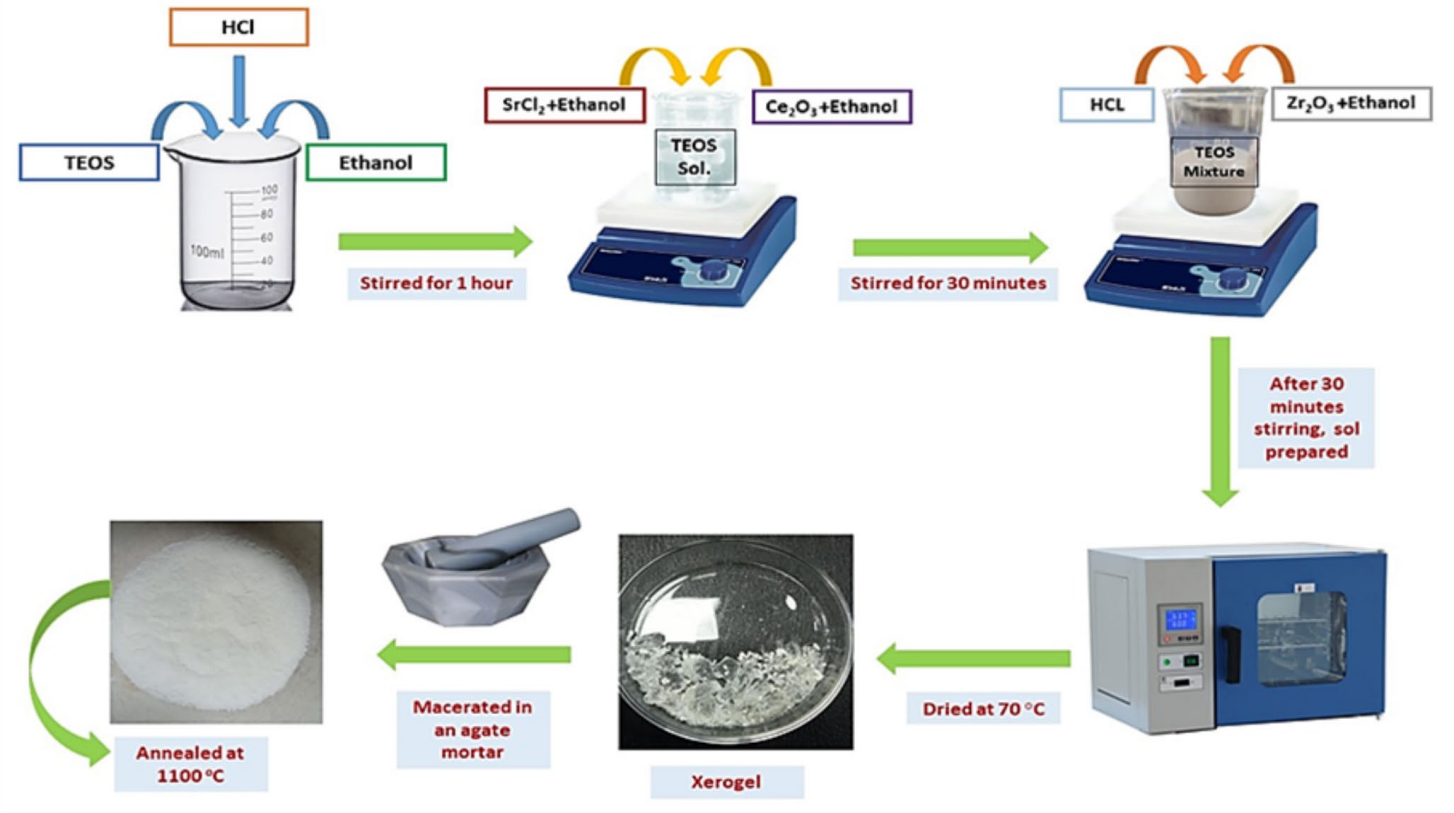
Schematic illustration of Ce^3+^ doped SiO_2_–Zr_2_O_3_:Sr^2+^ Sample Synthesis

High-purity chemicals and reagents were utilized in the synthesis of the NCs. These included crystal violet (C_25_H_30_ClN_3_-Sigma Aldrich 90%), curcumin (C_21_H_20_O_6_-Sigma Aldrich 98%), lycopene (C_40_H_56_-Sigma Aldrich 95%), ethanol (99.99%-Sigma Aldrich), hydrochloric acid (HCl- Synth-37%), cerium oxide (Ce_2_O_3_-Sigma Aldrich 99.99%), zirconium oxide (Zr_2_O_3_-Sigma Aldrich 99.99%), tetraethyl orthosilicate (TEOS-Sigma Aldrich 99.0%), SrCl_2_ (Sigma Aldrich 99.99%), ethanol (99.99%-Sigma Aldrich), hydrochloric acid (HCl-Synth-37%), and distilled water. To ensure the integrity and dependability of the experimental results, all chemicals and reagents were used exactly as received, without any additional purification. The synthetic scheme of Ce^3+^ doped SiO_2_, Zr_2_O_3_, and Sr^2+^ nanocomposite is illustrated in Fig. 1.

### Synthesis of Ce^3+^ Doped SiO_2_-Zr_2_O_3_: Sr^2+^ NCs

Ce^3+^ doped SiO_2_: Zr_2_O_3_: Sr^2+^ NCs were made by combining 3.8 milliliters of HCL (0.2 M) with 6 milliliters of ethanol and 11 milliliters of TEOS solution at room temperature and stirred for one hour. Then 0.223 g of Ce_2_O_3_ and 0.095 g of SrCl_2_ dissolved in 6 ml of ethanol separately, were transferred to the TEOS mixture, which was then rapidly stirred for an hour. In parallel zirconium oxide and ethanol were added at a 1:10 volume ratio (1 g of Zr_2_O_3_ in 9 ml of ethanol). The solutions containing TEOS and zirconium were mixed and left under stirring for 30 min. After mixing the two solutions, 1.0 ml of HCl (0.2 M) was added to the resultant mixture. The obtained sol was placed in a resistive oven and dried for several hours at 70 ^ο^C to remove the solvent and extract the material that would become the xerogel precursors. The xerogels were then obtained and macerated in an agate mortar. Safranin-O/Ce^3+^ doped SiO_2_: Zr_2_O_3_: Sr^2+^, CV/Ce^3+^ doped SiO_2_: Zr_2_O_3_: Sr^2+^, Curcumin/Ce^3+^ doped SiO_2_: Zr_2_O_3_: Sr^2+^, and Lycopene/Ce^3+^ doped SiO_2_: Zr_2_O_3_: Sr^2+^ samples were all prepared by following the procedure as mentioned above with safranin (1 ml), crystal violet (1 ml), curcumin (0.08 g), and lycopene (0.19 g). Only the Ce^3+^ doped SiO_2_: Zr_2_O_3_: Sr^2+^ sample was annealed at 1100 ^ο^C in a muffle furnace. Based on the results reported by Ferrari et al., [36] and as suggested by other studies [45] this temperature was chosen to achieve an ideal compromise between the crystallization and optical properties, improving thermal stability.

### Development of the Fingerprints

**Fig. 2:**
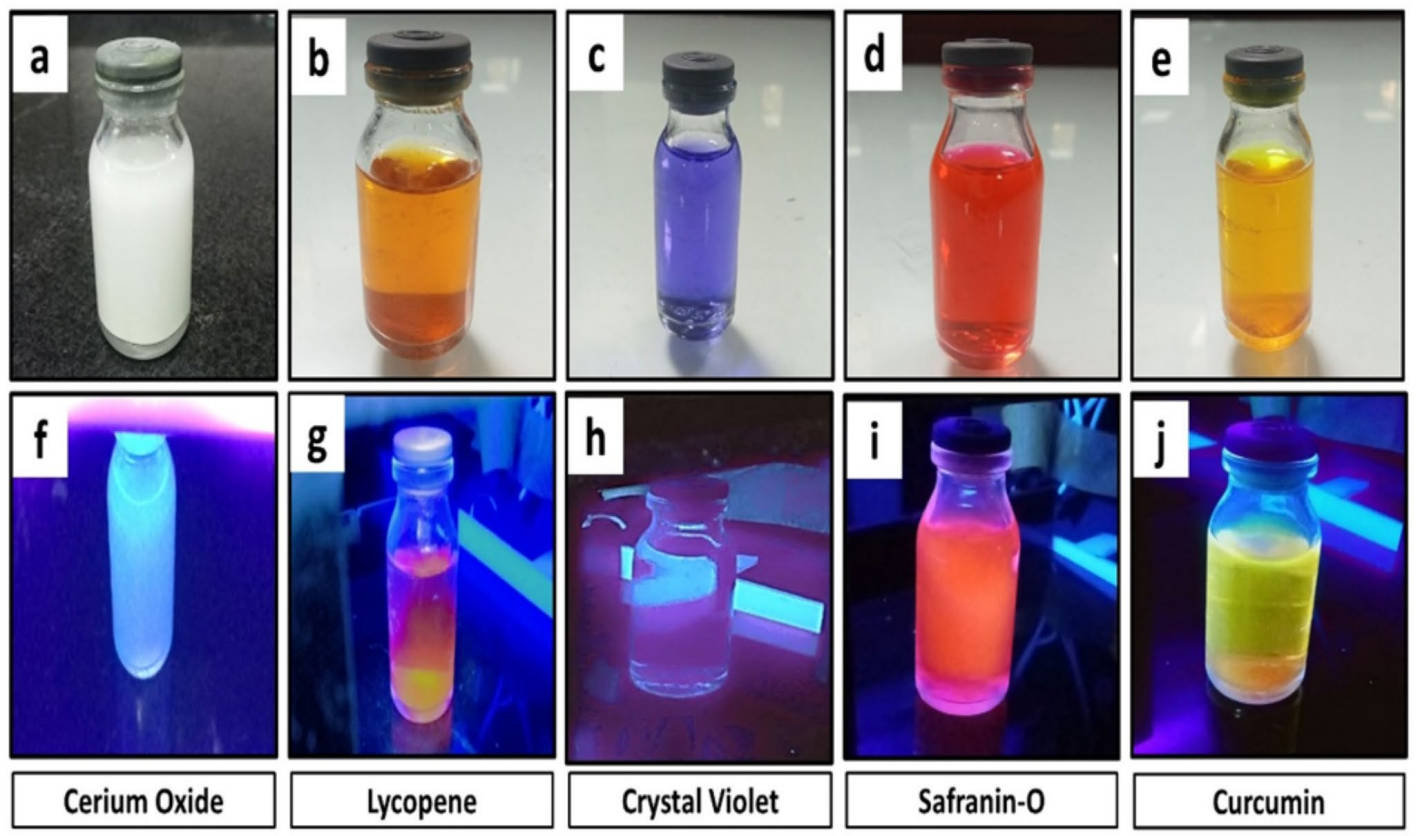
Photoluminescence of dyes in white light **(a-e)** and UV light **(f-j)**

The xerogels were macerated into a fine powder and then used to develop fingerprints. Using a magnetic brush, photoluminescent material was applied to the fingerprints on the slides. Then the excess powder was carefully removed with the brush, to avoid damaging the marked fingerprints. The slides with the identified fingerprints were placed in an ultraviolet (UV) chamber and subjected to a laser-assisted 379 nm excitation. This particular wavelength was carefully selected to ensure that the marked fingerprints would be easily identifiable in the resulting images. Finally, high-quality images of the marked fingerprints were taken using a smartphone camera. Figure 2 displays the photoluminescence of various pigments when exposed to UV light.

### Characterization Techniques

Powdered samples were analyzed using X-ray diffraction (XRD) using a Shimadzu diffractometer Model XRD 6000, CuKα: λ = 1.5418 Å, operated at 40 kV and 30 mA. Scanning electron microscopy (Hitachi S-4700 SEM) equipped with energy dispersive X-ray (EDX) was used to characterize the morphology and elemental analysis of the composite materials. The samples were loaded on carbon tape and gold sputtering was done at 1.2 to 1.5 kV for 30 s. DLS measurements were done to establish particle size dissemination (MALVERN Mastersizer 3000 + Ultra Instrument Limited, Worcestershire, United Kingdom). All the samples were prepared in DDW and filtered with a 0.45 µM nylon syringe filter. Photoluminescence (PL) emission intensities were recorded with a Horiba FluoroMax Spectrofluorometer (HORIBA Ltd. Japan) using a photomultiplier tube (PMT) detector.

## Results and Discussions

### XRD Analysis

**Fig. 3:**
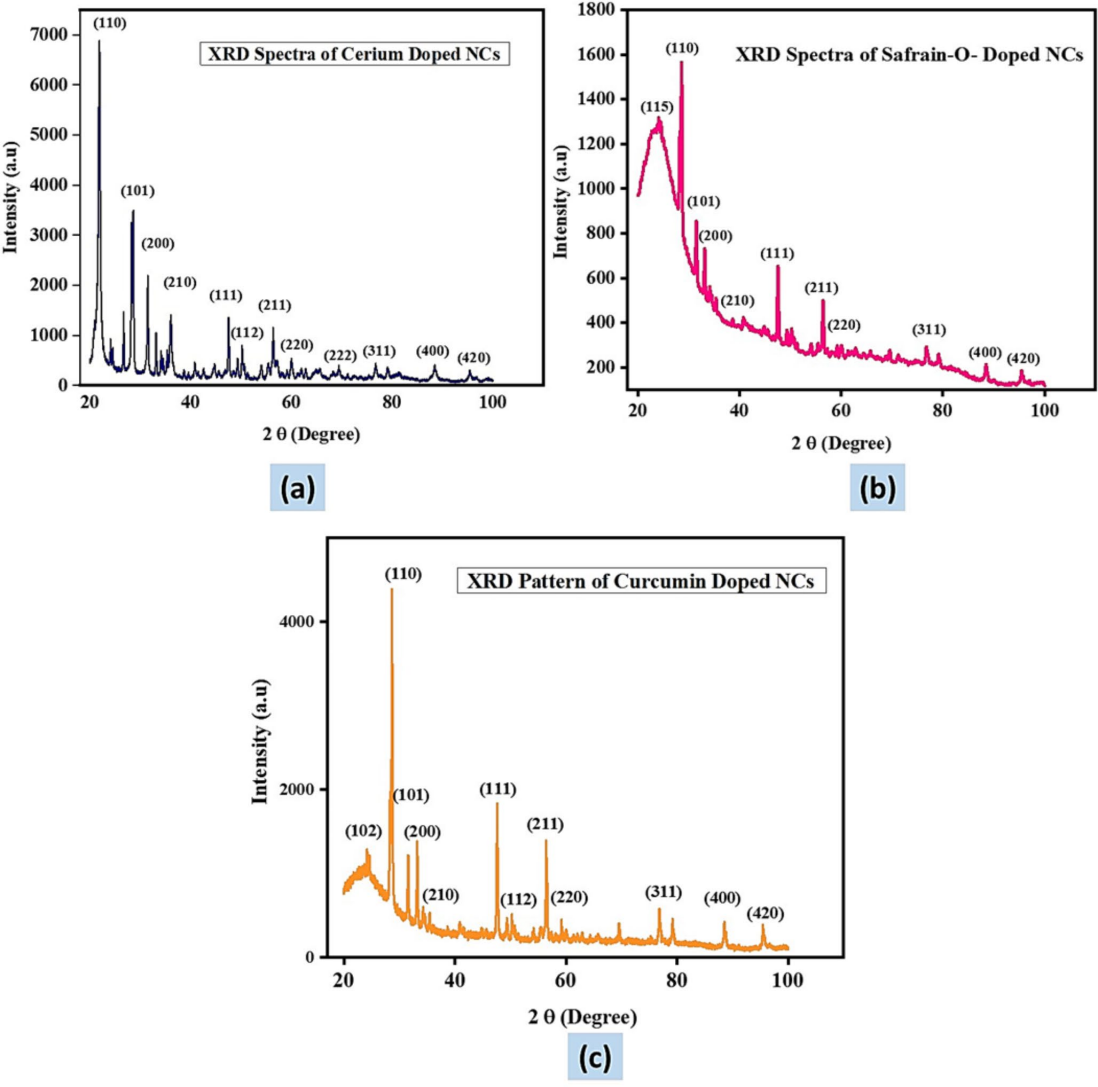
XRD pattern of Ce^3+^ doped SiO_2_-Zr_2_O_3_: Sr^2+^**(a)**, Safranin-O/Ce^3+^ doped SiO_2_-Zr_2_O_3_: Sr^2+^**(b)**, curcumin/Ce^3+^ doped SiO_2_-Zr_2_O_3_: Sr^2+^**(c)**

The nanocomposites under investigation were analyzed using X-ray diffraction to determine the crystal phases present within the material. The XRD patterns of Ce^3+^ doped SiO_2_-Zr_2_O_3_: Sr^2+^ (Fig. 3a), Safranin-O/Ce^3+^ doped SiO_2_-Zr_2_O_3_: Sr^2+^ (Fig. 3b) and curcumin/Ce^3+^ doped SiO_2_-Zr_2_O_3_: Sr^2+^ (Fig. 3c), were observed in the range of 20^ο^–100^ο^. The XRD analysis of the materials showed sharp diffraction peaks, making it possible to identify the prevailing crystal phases precisely. The region below 2θ = 30 ^ο^, showed a broad peak with a crystal facet (110), characteristic of amorphous materials. This intense peak is attributed to the presence of SiO_2_ in the system in this work, providing important information on the material’s composition. In the XRD pattern, peaks that developed at 2θ values of 30.4°, 35.3°, 50.5°, 59.5°, and 60.4°, are manifestations of the (101), (200), (210), (112), (211), (220) crystal facets that correspond to the tetragonal phase of Zr_2_O_3_ and match well with the ICCD card no (01-079-1764) [46]. The peaks positioned at 44.64°, 65.03°, 78.08°, 88.03°, and 95.12° are ascribed to the impurities, that is the sample holder’s chemical makeup as determined by XRD measurements. The absence of additional peaks such as SrO, Ce_2_O_3_, dyes, or other contaminants, indicates the formation of the pure material, further validating the accuracy of the analysis.

Peaks in the material network are slightly displaced towards higher angles as a result of microstrain created by the ion replacement process [47]. The displacement might be attributed to either the addition of Sr^2+^ to the material composition because of its large ionic radii (1.040 Å) relative to Zr^3+^ (0.80 Å) or a minor deformation of the symmetry site produced by inherent flaws [48]. Curcumin and Safranin-O doped materials showed minor distortion from the symmetry, causing the angle to shift to a slightly higher value, specifically from 21^ο^ to 24^ο^ due to the additional dye dopants that introduce mere defects into the crystal structure. This addition tends to increase the stability of the material, providing crucial insights into the material’s properties.

### EDX Analysis

**Fig. 4:**
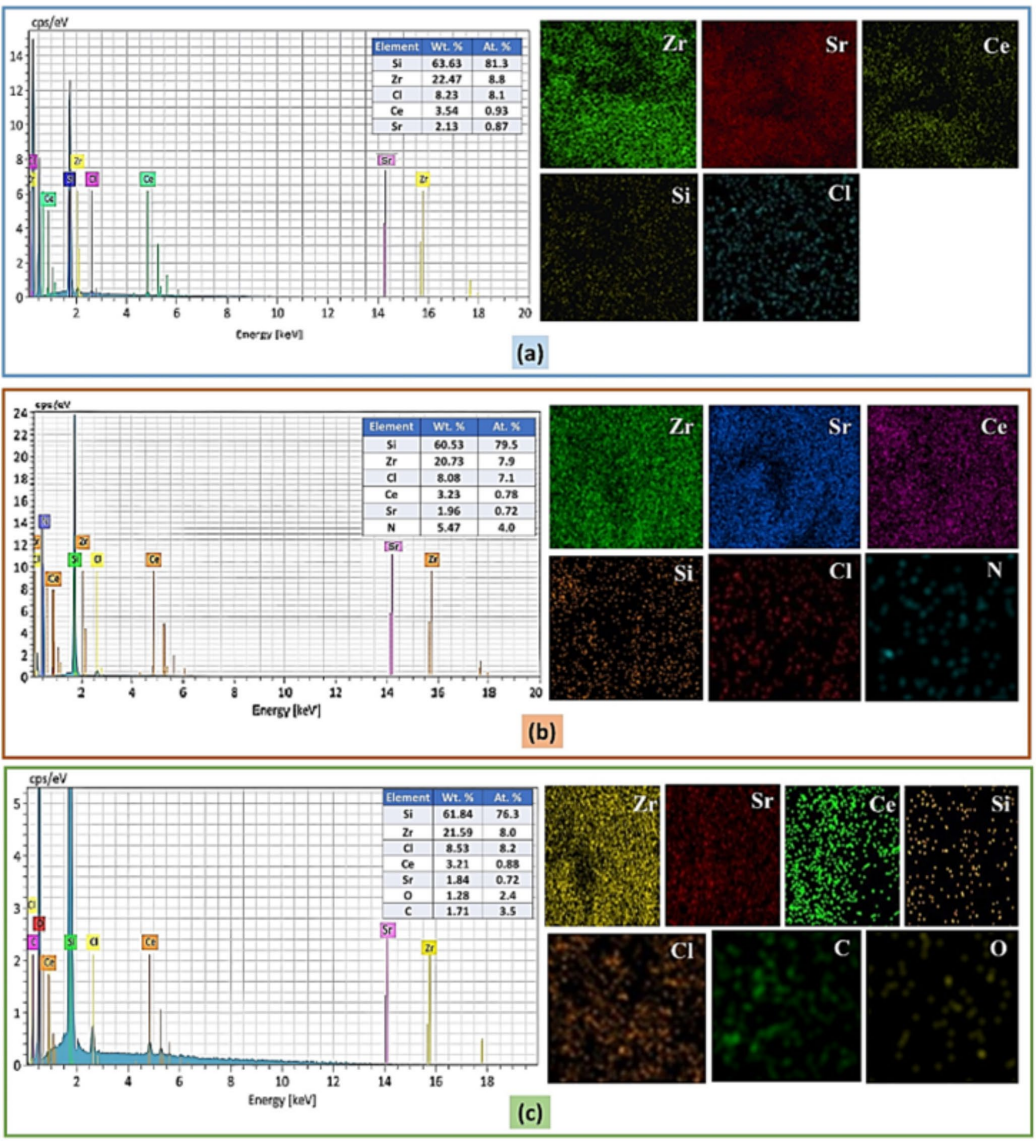
EDX spectra and mapping analysis of Ce^3+^ doped SiO_2_-Zr_2_O_3_: Sr^2+^**(a)**, Safranin-O/Ce^3+^ doped SiO_2_-Zr_2_O_3_: Sr^2+^**(b)**, Curcumin/Ce^3+^ doped SiO_2_-Zr_2_O_3_: Sr^2+^**(c)**

Figure 4**(a-c)** displays the EDX spectra of Ce^3+^ doped SiO_2_-Zr_2_O_3_: Sr^2+^, Safranin-O/Ce^3+^ doped SiO_2_-Zr_2_O_3_: Sr^2+^, and curcumin/Ce^3+^ doped SiO_2_-Zr_2_O_3_: Sr^2+^ samples, depicting the elemental composition. Additionally, the elemental percentage of each constituent was also computed using the EDX spectrum. For Ce^3+^ doped SiO_2_-Zr_2_O_3_: Sr^2+^ NCs (Fig. 4a) the distinct X-ray emissions confirmed the presence of various components, including strontium (Sr), silicon (Si), zirconium (Zr), chlorine (Cl), and cerium (Ce). Nitrogen (N) was confirmed in the Safranin-O/Ce^3+^ doped SiO_2_-Zr_2_O_3_: Sr^2+^ nanocomposite (Fig. 4b), along with cerium, silicon, zirconium, and strontium confirming the existence of Safranin-O. Additionally, carbon (C) and oxygen (O) peaks were found in the EDX pattern of the curcumin/Ce^3+^ doped SiO_2_-Zr_2_O_3_: Sr^2+^ nanocomposite (Fig. 4c), indicating the presence of curcumin. The peaks at 5–6 keV are consistent with the L-series emissions of cerium (L_β1_ = 5.26, L_β2_ = 5.61, and, Lγ = 6.05), and 17–18 keV could be associated with the K_β_ of zirconium (K_β_ = 17.67). Further, X-ray mapping analysis confirms the uniform distribution of the elements in the synthesized samples (Fig. 4a-c).

### SEM Analysis

**Fig. 5:**
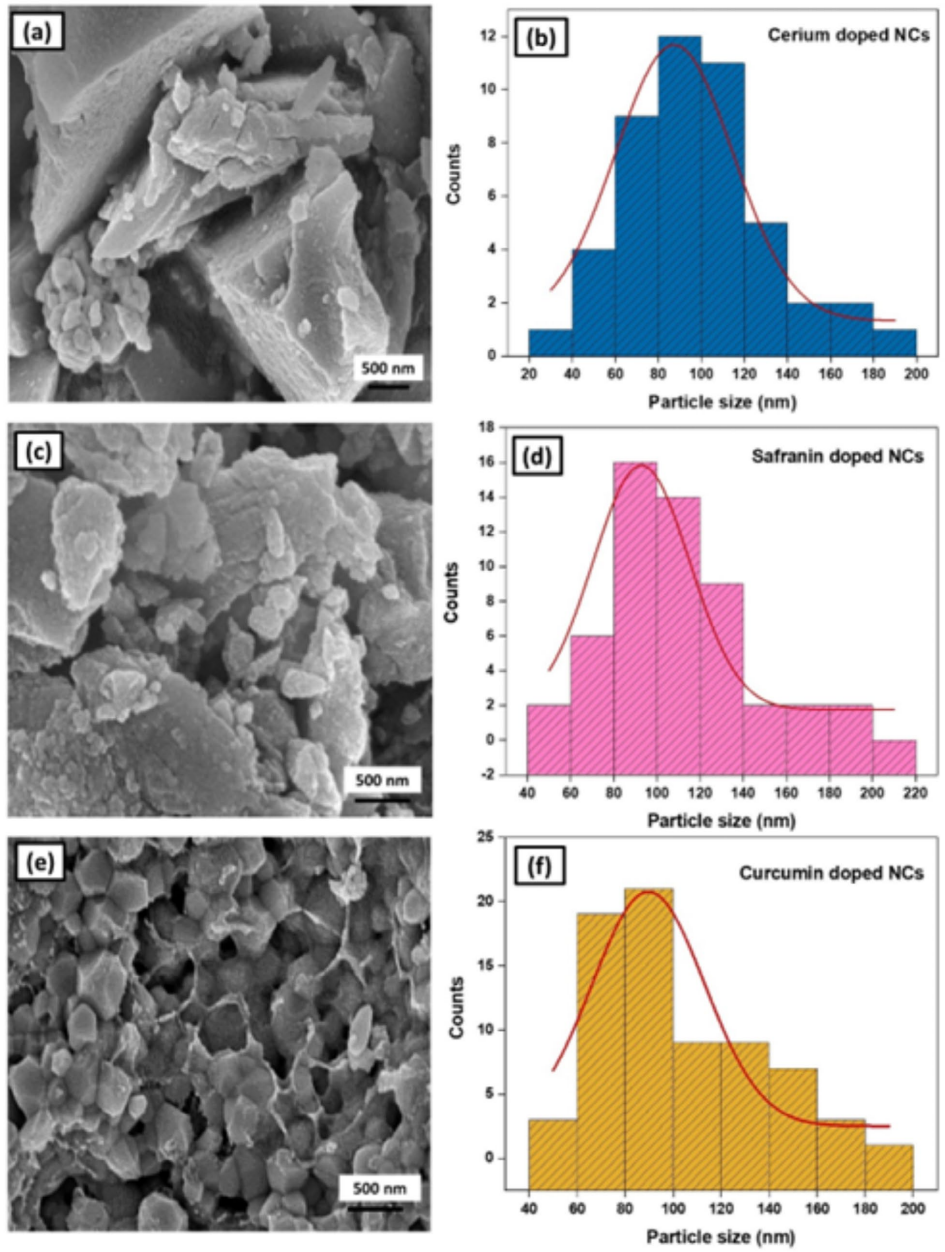
SEM profiles and particle size allocation histogram of Ce^3+^ doped SiO_2_-Zr_2_O_3_: Sr^2+^**(a**,** b)**, Safranin-O/Ce^3+^ doped SiO_2_-Zr_2_O_3_: Sr^2+^**(c**,** d)**, Curcumin/Ce^3+^ doped SiO_2_-Zr_2_O_3_: Sr^2+^**(e**,** f)**

The morphology and particle size distribution histograms of samples are displayed in Fig. 5. The SEM analysis of Ce^3+^ doped SiO_2_-Zr_2_O_3_: Sr^2+^ NCs (Fig. 5a**)** revealed uneven fractures, rough surfaces, and irregular groves with an average particle size of 87 nm. Safranin-O/Ce^3+^ doped SiO_2_-Zr_2_O_3_: Sr^2+^ (Fig. 5c**)** has a rough morphology with irregular particle size and shape, averaging about 92 nm. In the case of curcumin/Ce^3+^ doped SiO_2_-Zr_2_O_3_: Sr^2+^ SEM images exhibited a granular morphology with both fine and coarse particles as depicted in Fig. 5e with an average particle size of 89 nm. Agglomerations are observed, suggesting possible particle-particle interactions. The irregular shapes and particle sizes are ascribed to the intentional introduction of the dopants to create defects and enhance stability. These unique structural characteristics and customized chemical makeup make these NCs promising for various applications.

### DLS Analysis

**Fig. 6:**
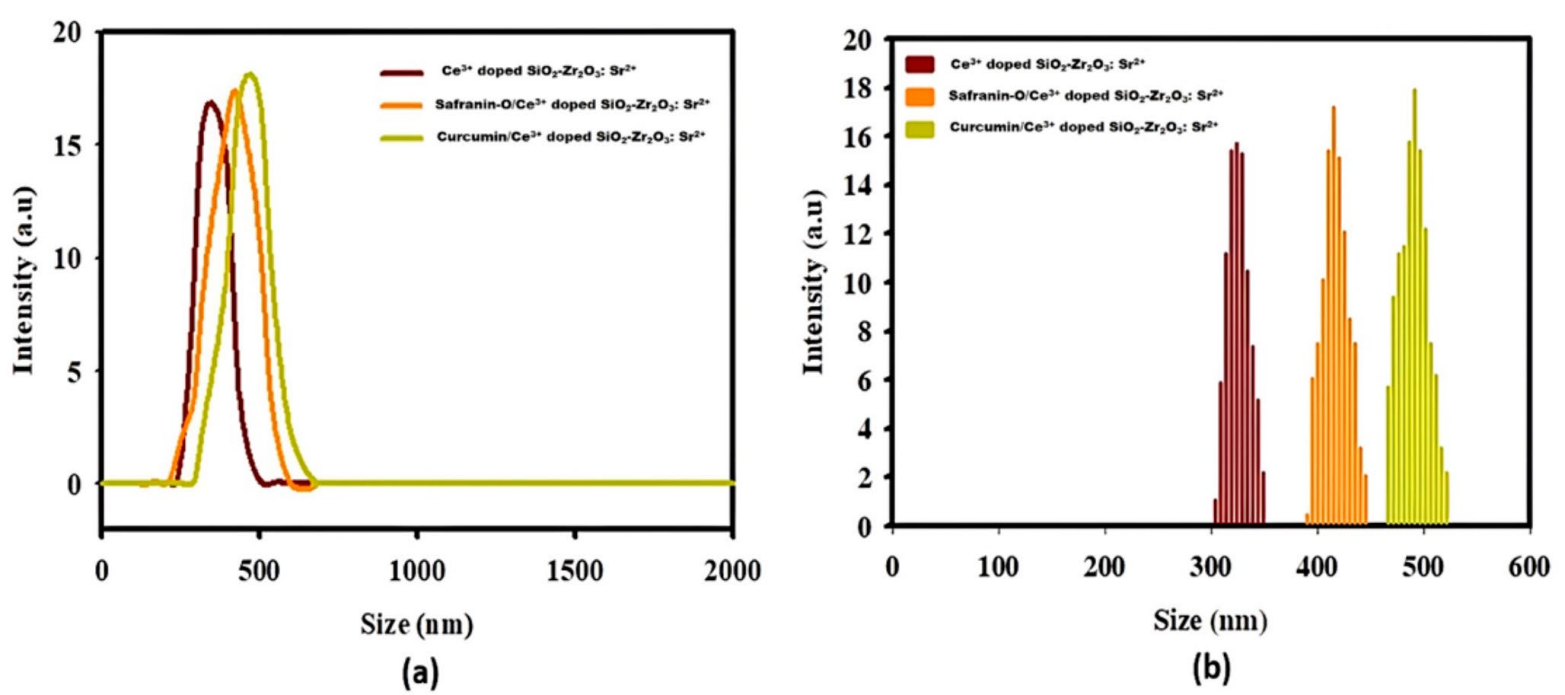
Curcumin/Ce^3+^ doped SiO_2_-Zr_2_O_3_: Sr^2+^ (green), Safranin-O/Ce^3+^ doped SiO_2_-Zr_2_O_3_: Sr^2+^ (orange), and Ce^3+^ doped SiO_2_-Zr_2_O_3_: Sr^2+^ (purple) particle size distribution by DLS. (**a**) The distribution curves (**b**) Distribution histogram

The hydrodynamic particle size distribution curve and histogram of nanocomposites were determined in aqueous solution using DLS measurements as shown in Fig. 6. DLS is an effective, non-invasive method for examining the characteristics and behavior of particles in a solution. It can yield useful data for various scientific and industrial applications. DLS produces images by using the Brownian motion of particles suspended in the solution. Because of their random mobility, the particles in the suspension generate variations in the strength of spread light when a laser beam passes through them. The behavior of our synthesized material (Ce^3+^ doped SiO_2_-Zr_2_O_3_: Sr^2+^) exhibited an average hydrodynamic diameter of approximately 321 nm. The particle size increased to around 410 nm in Safranin-O/Ce^3+^ doped SiO_2_-Zr_2_O_3_: Sr^2+^. Similarly, the curcumin/Ce^3+^ doped SiO_2_-Zr_2_O_3_: Sr^2+^ nanocomposite showed an increase in particle size to about 495 nm, providing clear details of the successful synthesis of nanocomposites.

### PL Analysis

**Fig. 7:**
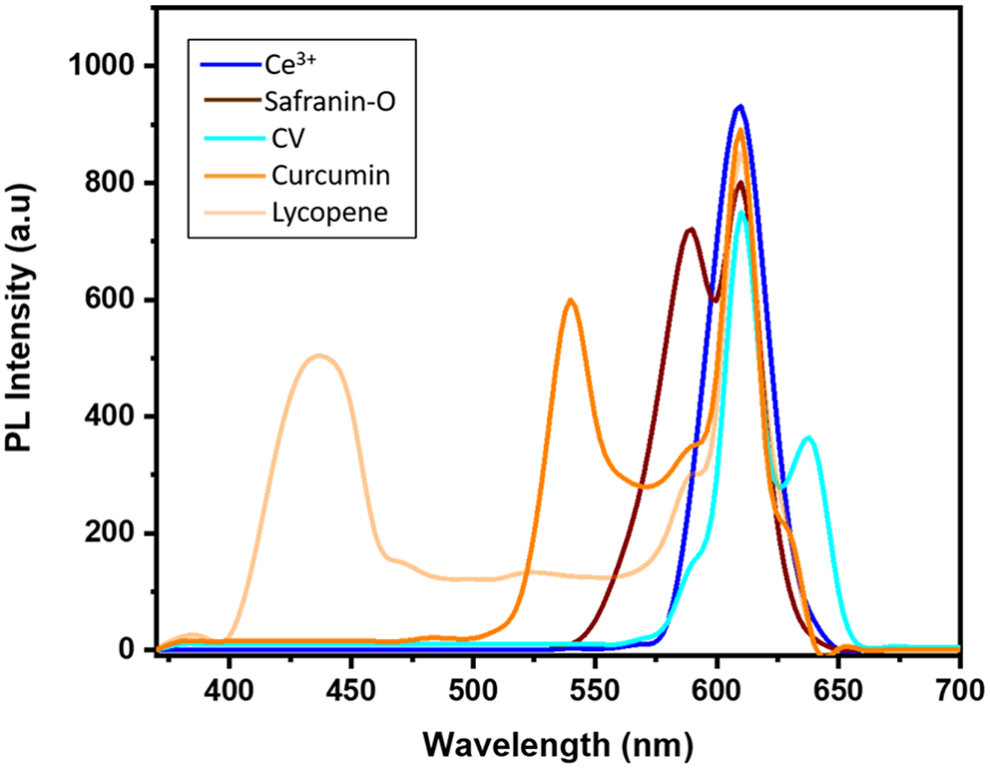
Comparison plot of photoluminescence intensities of Ce^3+^ doped SiO_2_-Zr_2_O_3_: Sr^2+^, Safranin-O/Ce^3+^ doped SiO_2_-Zr_2_O_3_: Sr^2+^, CV/ Ce^3+^ doped SiO_2_-Zr_2_O_3_: Sr^2+^, Curcumin/Ce^3+^ doped SiO_2_-Zr_2_O_3_: Sr^2+^, and Lycopene/Ce^3+^ doped SiO_2_-Zr_2_O_3_: Sr^2+^ NCs

Figure 7 presents a comparison of the PL intensities of all the samples. The measurements were made within the 370–700 nm range at room temperature. The emission spectra of the Ce^3+^ doped SiO_2_-Zr_2_O_3_: Sr^2+^ displayed a sharp, strong, and intense emission in the red region at 620 nm. Two peaks could be seen in the emission spectra of the sample doped with Safranin-O, one at 620 nm and the other at 587 nm. The peak at 587 nm was attributed to the presence of the Safranin-O which is its characteristic peak as reported in the literature [49]. Similarly, the sample doped with crystal violet al.so showed two peaks, one around 636 nm characteristic of the CV dye, and the peak due to the presence of Ce^3+^. In curcumin doped sample the curcumin emission peak was recorded at 543 nm and for lycopene around 430 nm (since lycopene is a weak photoluminescent dye) in addition to the peak of Ce^3+^ at 620 nm that was found in all the samples.

This intense red emission is attributed to the presence of the Ce^3+^ ions making this material suitable for developing high-resolution fingerprints for forensic applications. However, in the case of samples doped with Safranin-O, crystal violet [50], curcumin, and lycopene the reduced intensity of Ce^3+^ was observed. Ultimately, it can be concluded that the dye doping diminished both the intensity and the clarity of the photoluminescent emission. Fluorescence intensity quenching can result from various molecular interactions within the formed crystal. Molecular reorganizations, ground state complex development, transfer of energy, excited state processes, and collisional quenching are a few examples of potential molecular interactions [51, 52]. However, it was observed that due to the addition of dyes as dopants, the peak got narrower in this case, and was attributed to the change in the situation around the Ce^3+^ ions that might lead to the modification of energy levels resulting in smoother and more precise energy transitions, but the intensities of the peaks were compromised.

**Fig. 8:**
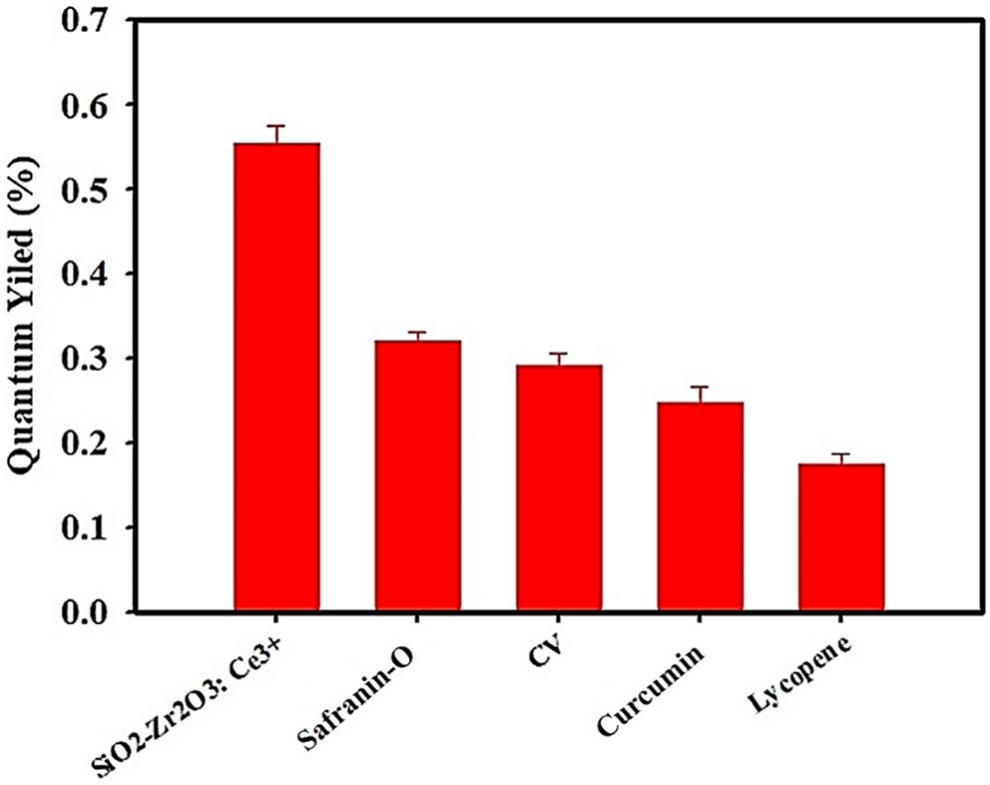
Quantum yield measurements of Ce^3+^ doped SiO_2_-Zr_2_O_3_: Sr^2+^, Safranin-O/Ce^3+^ doped SiO_2_-Zr_2_O_3_: Sr^2+^, CV/ Ce^3+^ doped SiO_2_-Zr_2_O_3_: Sr^2+^, Curcumin/Ce^3+^ doped SiO_2_-Zr_2_O_3_: Sr^2+^, and Lycopene/Ce^3+^ doped SiO_2_-Zr_2_O_3_: Sr^2+^ NCs

In addition, the quantum yield Φ of the samples (Fig. 8) was also calculated by using the formula.

$$\:\varPhi\:=\:{\varPhi\:}_{R}\times\:\frac{I}{{I}_{R}}\times\:\frac{{A}_{R}}{A}\times\:\frac{{\eta\:}^{2}}{{\eta\:}_{R}^{2}}$$

Where Φ is the quantum yield of the sample and Φ_R_ is the quantum yield of the reference (CQDs in this case). I and I_R_ are the integrated fluorescence intensities, A and A_R_ are the absorbance at the excitation wavelength, n and n_R_ are the refractive indices of the solvent used for the sample and reference correspondingly. The refractive index correction was implemented because the solvents in which the sample and the reference were dissolved had distinct refractive indices, ensuring the accuracy of the quantum yield measurements. Ce^3+^ doped SiO_2_-Zr_2_O_3_: Sr^2+^ showed the highest quantum yield (0.58% ± 0.1) among other samples.

### Ce^3+^ Concentration Optimization Studies

**Fig. 9:**
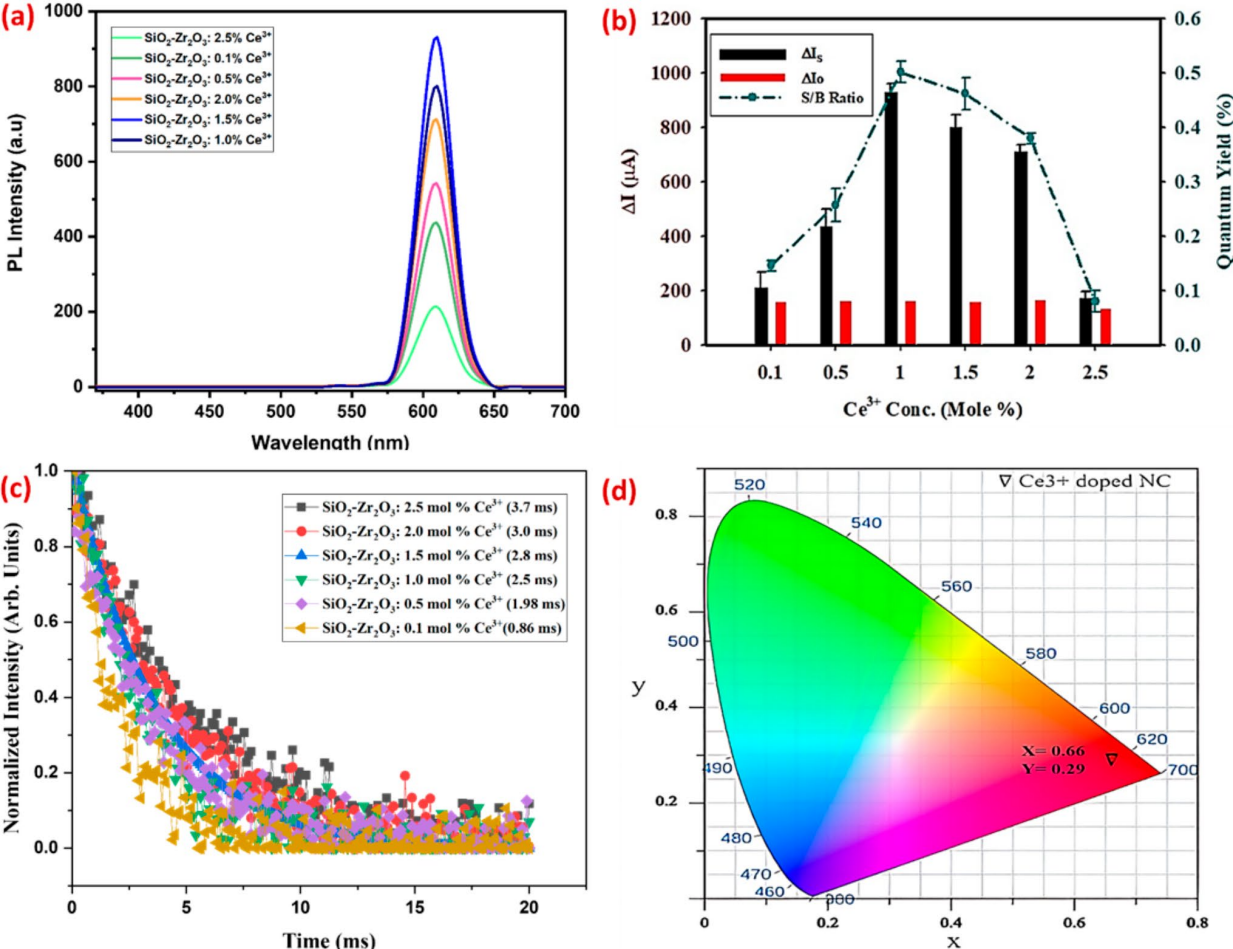
PL emission spectra of xCe^3+^ doped SiO_2_-Zr_2_O_3_: Sr^2+^ samples **(a)** quantum yields of xCe^3+^ doped SiO_2_-Zr_2_O_3_: Sr^2+^ samples **(b)** fluorescence lifetime curves xCe^3+^ doped SiO_2_-Zr_2_O_3_: Sr^2+^ samples **(c)** CIE chart diagram of 1 mol% Ce^3+^ doped SiO_2_-Zr_2_O_3_: Sr^2+^ sample **(d)**

Ce^3+^-doping concentration optimization was also carried out to understand the influence of doping concentration on photoluminescent properties (Fig. 9). A series of xCe^3+^ doped SiO_2_-Zr_2_O_3_: Sr^2+^ (x = 0.1, 0.5, 1.0, 1.5, 2, 2.5 mol%) samples were synthesized for PL optimization studies. Figure 9a shows that the Ce^3+^-doping concentration does not affect emission spectra however the PL intensities of the samples increase with increasing Ce^3+^ concentration reaching a maximum at about 1 mol% (Fig. 9b). Then due to the concentration quenching behavior, the intensity begins to decline gradually. The fluorescence lifetime decay curves (Fig. 9c) of xCe^3+^ doped SiO_2_-Zr_2_O_3_: Sr^2+^ (x = 0.1, 0.5, 1.0, 1.5, 2, 2.5 mol%) nanocomposites were measured by exciting the samples with wavelength 379 nm with emission peaks observed at 620 nm adjusted in accordance to the second order exponential decay by using the following equation [53]:

$$\:{I=I}_{o}\left({A}_{1}{{exp}}^{-t/\tau\:1}\:{+\:A}_{2}{exp}^{-t/\tau\:2}\:\right)$$

Where, I is the intensity that will decay with time, I_ο_ is the initial intensity at time t = 0. A_1_ and A_2_ are the fitting parameters. τ_1_ and τ_2_ are the average lifetime values.

The calculated average lifetime values of Ce^3+^ ions are presented in Table 1. The values are consistent with the literature [54, 55]. With 2.5 mol% Ce^3+^ concentrations, the lifetime decay curves displayed a longer value of 3.7 ms, and with 0.1% mol% Ce^3+^ concentrations, the lowest value of 0.86 ms. A progressive rise in lifespan values was seen when Ce^3+^ concentrations rose, maybe as a result of higher concentrations of emission energy being absorbed [56]. 2.5 mol% shows intense quenching despite having the longest lifetime decay. Thus, 1 mol% Ce^3+^ doping concentration corresponding to the amount taken for preparing the nanocomposites in this study was considered promising for fingerprinting applications with milliseconds lifetime emission values (2.5 ms) and intense fluorescent emissions.

**Table 1:** Average lifetime values of Ce^3+^ in SiO_2_-Zr_2_O_3_: Sr^2+^

Sample ID	Average lifetime (ms)
SiO_2_-Zr_2_O_3_: 0.1 mol %Ce^3+^	0.86
SiO_2_-Zr_2_O_3_: 0.5 mol %Ce^3+^	1.98
SiO_2_-Zr_2_O_3_: 1.0 mol %Ce^3+^	2.5
SiO_2_-Zr_2_O_3_: 1.5 mol %Ce^3+^	2.8
SiO_2_-Zr_2_O_3_: 2.0 mol %Ce^3+^	3.0
SiO_2_-Zr_2_O_3_: 2.5 mol %Ce^3+^	3.7

Figure 9d depicts the CIE diagram of our synthesized Ce^3+^ doped SiO_2_-Zr_2_O_3_: Sr^2+^ nanocomposite with optimal doping concentration (1 mol%). Commission Internationale de I’Eclairage (CIE) color coordinates are used to characterize and determine the proper color of luminescence, indicating the color purity of the material. CIE 1931 color chromaticity coordinates (xy) of the 1 mol% Ce^3+^ doped SiO_2_-Zr_2_O_3_: Sr^2+^ were determined by using the PL emission data. According to the CIE chart, the chromaticity coordinates of the material lie in the red region of the chart diagram.

### Latent Fingerprints Identification

**Fig. 10:**
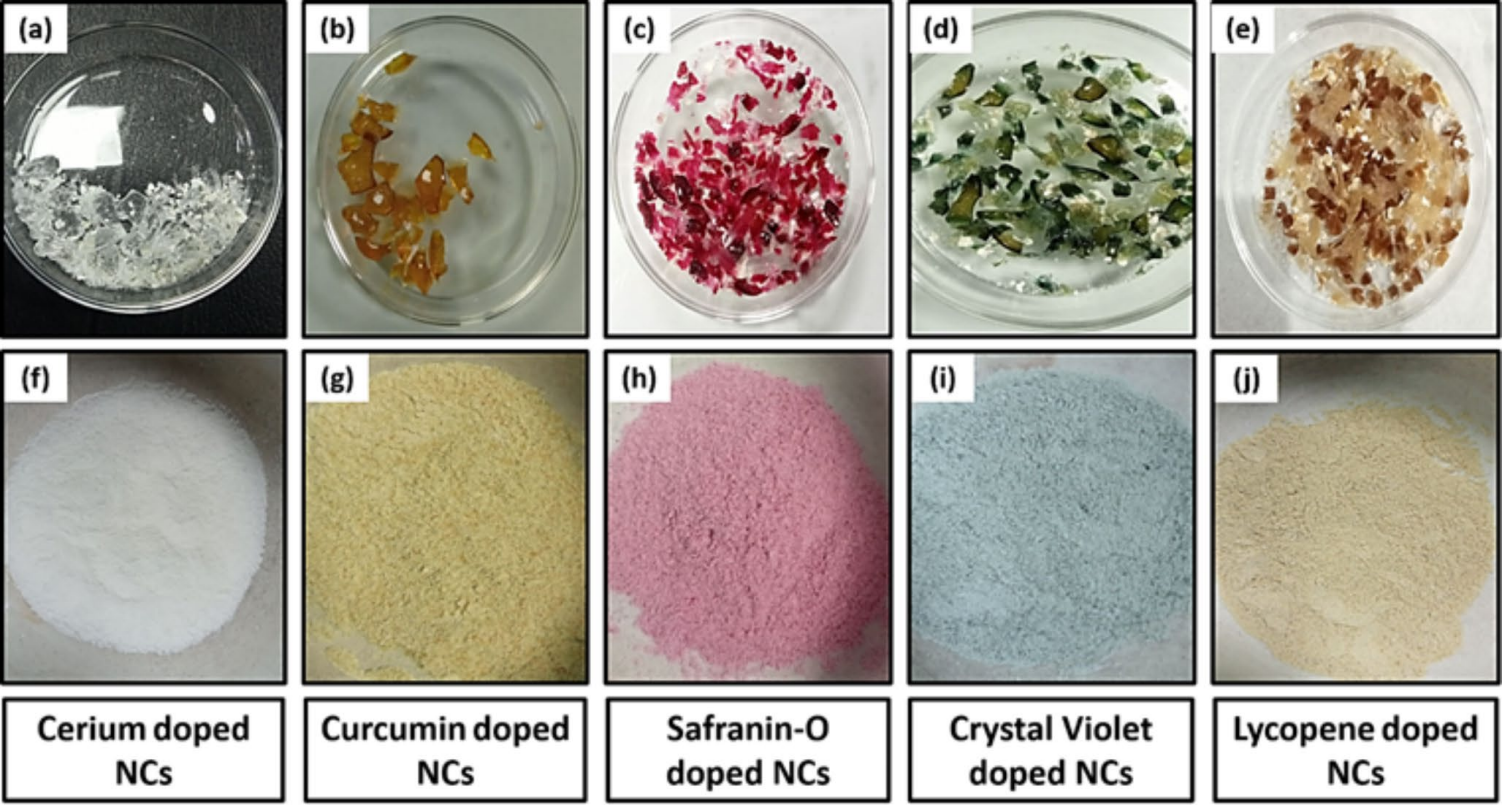
Xerogels **(a-e)**, crushed into nanocomposite powder (**f-j)**

Xerogels were crushed into a finely ground powder for easy application on surfaces as shown in Fig. 10. Because of their distinctive characteristics, silicon oxide nanoparticles are often employed for fingerprint development. These nanoparticles can interact with the oils, residues, humidity sweat, and moisture found in fingerprints due to their large surface area [57]. By adhering to the ridges of the fingerprint and producing a contrast between them and the surface, the particles increase the fingerprint’s visibility. Since silicon oxide nanoparticles are versatile, non-toxic, environmentally friendly, optically transparent, and stable, they are a great option for fingerprint development [58]. They can be used against various colored surfaces when paired with photoluminescent materials.

**Fig. 11:**
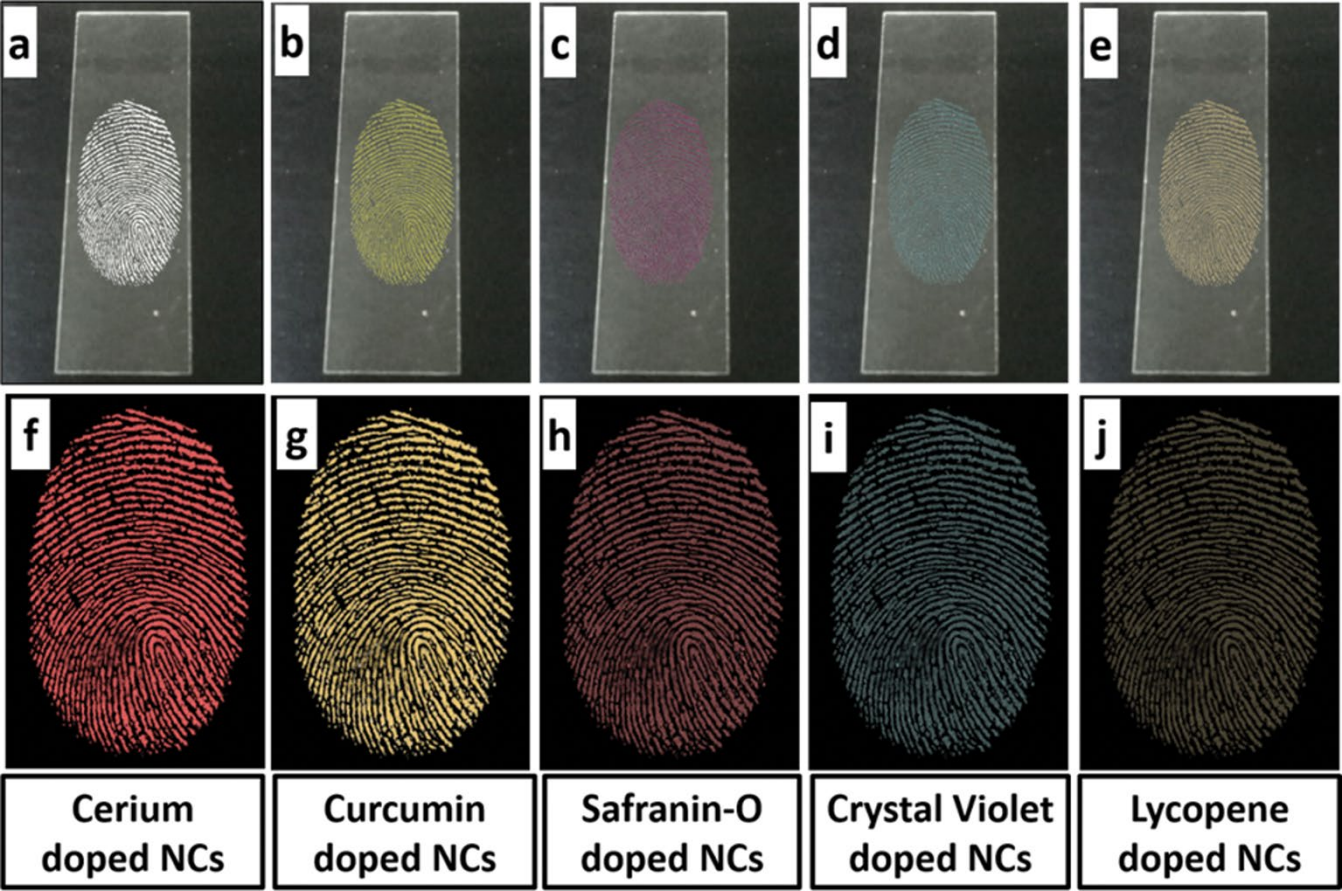
Fingerprints developed with variants of nanocomposites under white light **(a-e)**, and under UV light (379 nm) (**f-j)**

All the samples showed good adhesiveness to the fingerprint material on the glass slide. Ce^3+^ doped SiO_2_-Zr_2_O_3_: Sr^2+^ and curcumin/Ce^3+^ doped SiO_2_-Zr_2_O_3_: Sr^2+^ materials showed the highest visibility and clarity against the dark background. And when exposed to UV light they showed bright luminescence among all the samples as shown in Fig. 11. Significant research has been published on developing fingerprint dusting powders with luminescent properties. For this purpose, several dyes and powders have been investigated and our study was consistent with earlier research. The best NCs among all those prepared samples are thought to be Ce^3+^ doped SiO_2_-Zr_2_O_3_: Sr^2+^ as they showed great luminescence and exceptional stability. Most notably, it doesn’t include any of the harmful dyes that are conventionally employed, hence no dye is required to make the material photoluminescent.

### Ridge Pattern Study of Developed Fingerprints

**Fig. 12:**
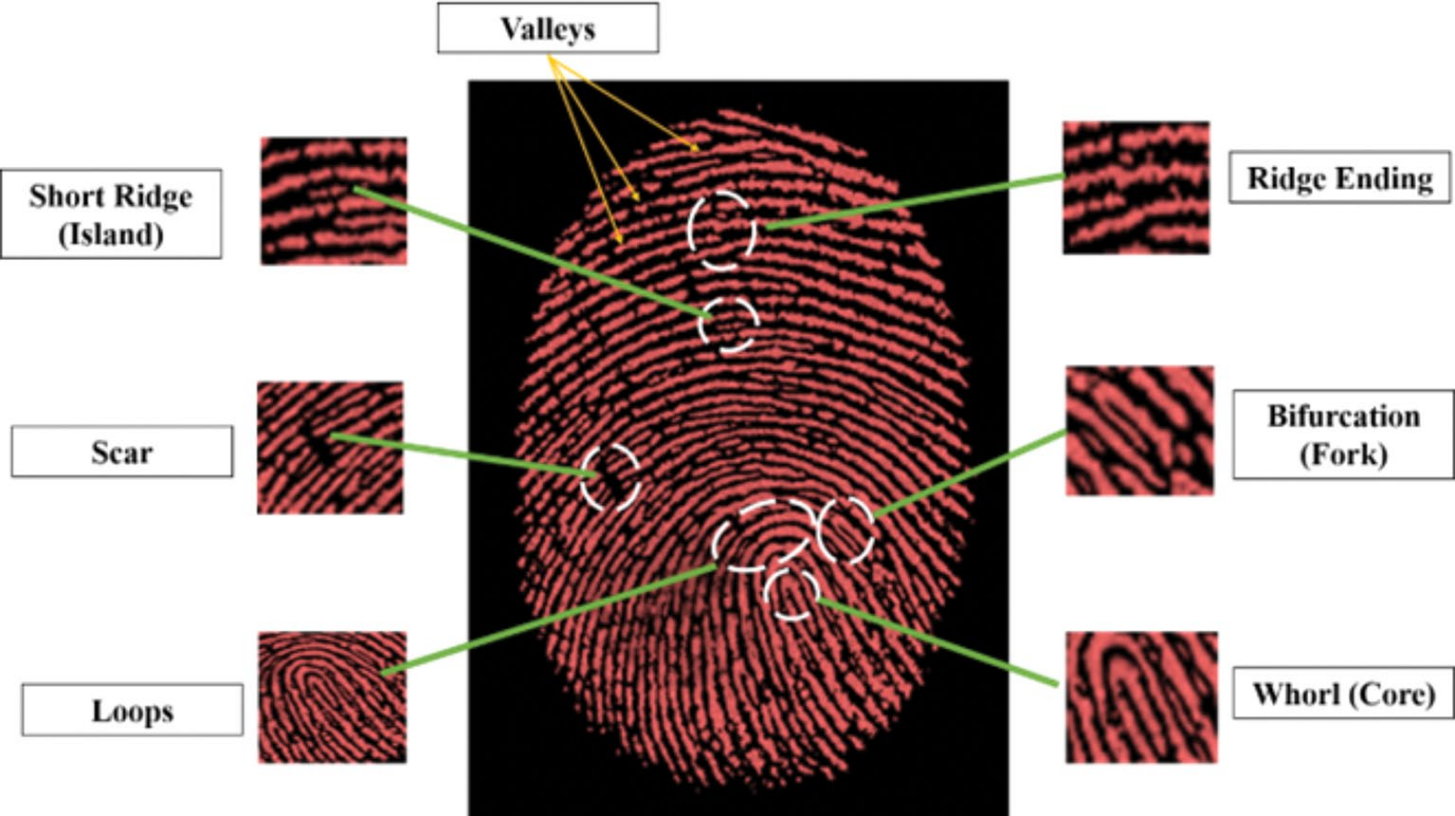
Different ridge patterns of LFPs visualized by Ce^3+^ doped SiO_2_-Zr_2_O_3_: Sr^2+^ nanocomposite

The second stage ridge pattern details were revealed by the developed fingerprints including bifurcation (fork), valleys, scar, short ridge (island), loops, whorl (core), and ridge ending as displayed in Fig. 12. The ridge details and pattern can help identify the suspects. Thus the synthesized nanocomposite powders demonstrated high sensitivity and validity for latent fingerprint development and identification, proving to be an effective labeling agent with intense red emission and high thermal stability.

## Conclusion

The sol-gel method made it simple to create a material based on SiO_2_-Zr_2_O_3_: Ce^3+^, Sr^2+^. The material was further co-doped with naturally fluorescent dyes including Safranin-O, crystal violet, curcumin, and lycopene. The yield was excellent, and the synthesis technique was straightforward and effective. All the samples showed good adhesiveness with the fingerprint material. All of the samples’ analyses revealed the presence of a narrow band in the spectrum at about 620 nm, which is attributed to the Ce^3+^ positioned inside the crystalline structure. Although the stability of dye-doped samples was improved, the dyes still couldn’t withstand extremely high temperatures. The cerium-doped material (1 mol %) is considered the best among all samples due to its strong photoluminescence in the red region of the spectrum. Because of the pigments they contain, NCs doped with synthetic dyes are highly hazardous to the user, and those doped with both synthetic and organic dyes were not tolerant to extreme temperatures. While analyzing Ce^3+^ doped SiO_2_-Zr_2_O_3_: Sr^2+^ nanocomposite it’s important to note that the annealing at 1100 ^ο^C enhanced the material’s optical properties and thermal stability. If you treat this material at the same temperature to remove any organic residue that might become embedded in it while fingerprints are being marked, you can retrieve and reuse it. This was not possible with dye-doped samples. Thus, considering the following: the material’s robustness, heat or light resistance of the xerogels, relatively simple preparation, high quantum yield, facile impregnation of photoluminescent materials on the fingerprints’ surface, reusability, and most importantly, non-toxic nature, the Ce^3+^ doped SiO_2_-Zr_2_O_3_: Sr^2+^ material is an optimistic system to be used as a fingerprint marker at the sites of crime as well as in other areas of forensics.

## Data Availability

The datasets generated during and/or analyzed during the current study are available from the corresponding author upon reasonable request.
